# Supplementary material for: Integrated analysis of the aqueous humor microbiome and lens capsule transcriptome in high myopia cataract: a pilot study
Source: Front Med (Lausanne). 2026 Jun 16;13:1845205. doi: 10.3389/fmed.2026.1845205 (PMC13314463; doi:10.3389/fmed.2026.1845205)
Supplement: Supplementary file 3 [file Data_Sheet_1.zip › 4.Meta_Statistic/C372089/LEfSe/biomarkers_raw_images/1_f__Enterobacteriaceae.pdf]

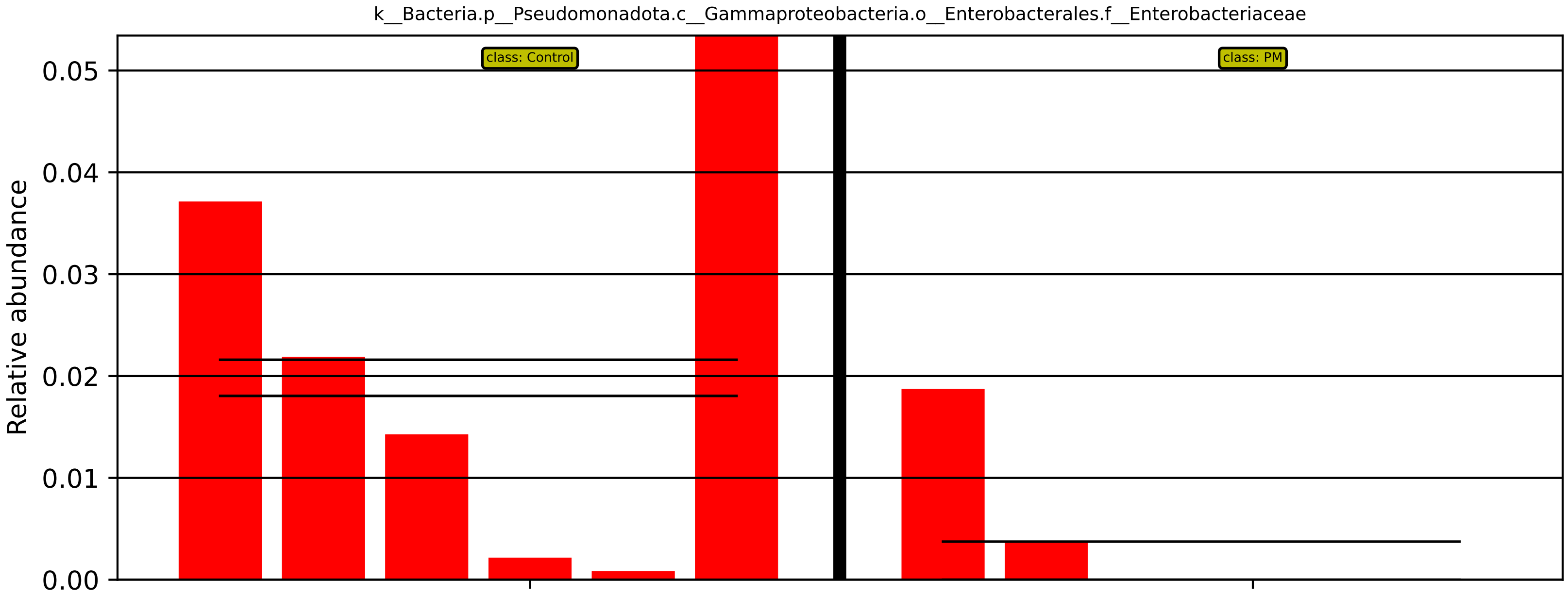

k__Bacteria.p__Pseudomonadota.c__Gammaproteobacteria.o__Enterobacterales.f__Enterobacteriaceae
class: Control
class: PM
Relative abundance
0.05
0.04
0.03
0.02
0.01
0.00
